# Deciphering the Arginine-Binding Preferences at the Substrate-Binding Groove of Ser/Thr Kinases by Computational Surface Mapping

**DOI:** 10.1371/journal.pcbi.1002288

**Published:** 2011-11-17

**Authors:** Avraham Ben-Shimon, Masha Y. Niv

**Affiliations:** Institute of Biochemistry, Food Science and Nutrition, The Robert H. Smith Faculty of Agriculture, Food and Environment and The Fritz Haber Center for Molecular Dynamics, The Hebrew University, Israel; University of California San Diego, United States of America

## Abstract

Protein kinases are key signaling enzymes that catalyze the transfer of γ-phosphate from an ATP molecule to a phospho-accepting residue in the substrate. Unraveling the molecular features that govern the preference of kinases for particular residues flanking the phosphoacceptor is important for understanding kinase specificities toward their substrates and for designing substrate-like peptidic inhibitors. We applied ANCHORSmap, a new fragment-based computational approach for mapping amino acid side chains on protein surfaces, to predict and characterize the preference of kinases toward Arginine binding. We focus on positions P−2 and P−5, commonly occupied by Arginine (Arg) in substrates of basophilic Ser/Thr kinases. The method accurately identified all the P−2/P−5 Arg binding sites previously determined by X-ray crystallography and produced Arg preferences that corresponded to those experimentally found by peptide arrays. The predicted Arg-binding positions and their associated pockets were analyzed in terms of shape, physicochemical properties, amino acid composition, and in-silico mutagenesis, providing structural rationalization for previously unexplained trends in kinase preferences toward Arg moieties. This methodology sheds light on several kinases that were described in the literature as having non-trivial preferences for Arg, and provides some surprising departures from the prevailing views regarding residues that determine kinase specificity toward Arg. In particular, we found that the preference for a P−5 Arg is not necessarily governed by the 170/230 acidic pair, as was previously assumed, but by several different pairs of acidic residues, selected from positions 133, 169, and 230 (PKA numbering). The acidic residue at position 230 serves as a pivotal element in recognizing Arg from both the P−2 and P−5 positions.

## Author Summary

Protein kinases are key signaling enzymes and major drug targets that catalyze the transfer of phosphate group to a phospho-accepting residue in the substrate. Unraveling molecular features that govern the preference of kinases for particular residues flanking the phosphoacceptor (substrate consensus sequence, SCS) is important for understanding kinase-substrates specificities and for designing peptidic inhibitors. Current methods used to predict this set of essential residues usually rely on linking between experimentally determined SCSs to kinase sequences. As such, these methods are less sensitive when specificity is dictated by subtle or kinase-unique sequence/structural features. In this study, we took a different approach for studying kinases specificities, by applying a new fragment-based method for mapping amino acid side chains on protein surfaces. We predicted and characterized the preference of Ser/Thr kinases toward Arginine binding, using the unbound kinase structures. The method produced high quality predictions and was able to provide novel insights and interesting departures from the prevailing views regarding the specificity-determining elements governing specificity toward Arginine. This work paves the way for studying the kinase binding preferences for other amino acids, for predicting protein-peptide structures, for facilitating the design of novel inhibitors, and for re-engineering of kinase specificities.

## Introduction

Protein phosphorylation is one of the most abundant posttranslational modifications. It is catalyzed by protein kinases, a large group of enzymes that account for approximately 2% of the human genome [1]. Phosphorylation involves the regulation of almost every process in the cell, and numerous diseases, such as diabetes, Alzheimer's disease and cancer, are tightly related to abnormal levels of protein phosphorylation. Thus kinases are considered one of the major drug targets of the 21^st^ century [2], with over a hundred kinase inhibitors in various stages of clinical trials and several drugs already in the clinic [3].

Most kinase inhibitors target the ATP-binding site [4], providing different, but usually low levels of kinase selectivity [5]. In pursuit of additional (non-ATP site) ways of inhibiting kinases, which in some cases may provide kinase-selective inhibition [6], kinase-substrate and other kinase-protein interactions are being actively targeted by various research groups using small molecules [7], [8] and peptidomimetics [6], [9], [10], [11], [12]. Structural information and computational approaches have greatly contributed to the design of low-molecular-weight kinase-targeting drugs [13]. The need for computational tools for peptide design is on the rise, due to increasing interest in protein-protein interactions and their inhibition in general [11], [14], [15], [16] and for protein kinases in particular [6], [9], [17], providing part of the motivation for the current work. While peptides are usually considered poor drug candidates because of low cell permeability and high tendency to be rapidly metabolized, recent improvements in synthetic peptide chemistry [18], successful usage of modulations that enable cell-penetration of proteins and peptides [6], [12], [19], [20], [21], [22] and of different administration routes, open up new avenues in the field of peptidic and peptidomimetic drug discovery [23].

Members of the protein kinase family share a common structure, consisting of a small N-terminal lobe and a larger C-terminal lobe [24]. The ATP-binding site and the main substrate-recognition site lie within the major groove formed between the two lobes. In eukaryotes, most kinases transfer the ATP γ-phosphate to either serine or threonine residues (Ser/Thr kinases), while others phosphorylate tyrosine residues (Tyr kinases) [25]. The Ser/Thr kinases can be further classified into various families and subfamilies based on sequence similarity, such as ACG, CAMK, etc. [1]. Another common classification of Ser/Thr kinases is into three main groups, basophilic, acidophilic and proline-directed. This classification is based on basic, acidic or proline substrate residues that govern kinase-substrate recognition [26], [27], and is assumed to confer global specificity between the three groups of kinases [28].

The above-mentioned residues are part of a set of amino acid residues immediately flanking the substrate phosphorylation site (which is referred to as P0) that play an important role in the tendency of the substrate to be recognized and phosphorylated by a particular kinase. The term substrate consensus sequence (SCS) refers to the essential sequence elements surrounding the phosphorylated site [29]. The flanking residues are referred to as P−n or P+n according to their location along the substrate sequence, n residues N-terminal or C-terminal to the P0 position, respectively.

Early studies of the prototypical basophilic protein kinase A (PKA) showed a pronounced preference for Arginine (Arg) at positions P−2 and P−3 of the substrate [30]. Later, the strong preference for Arg at P−3 was shown to be a general feature of many basophilic kinases. In a recent work that tested the consensus phosphorylation motifs of 61 out of the 122 kinases in *Saccharomyces cerevisiae*, 57% were detected as basophilic, with 87% of the basophilic kinases showing a primary preference for Arg at the P−3 position [31]. In fact, peptide phosphorylation screening approaches often fix Arg at this position, and concentrate on exploring preferences at other positions [32].

Aside from the P−3 position, P−2 and P−5 are the only two positions for which the frequency of Arg is greater than its average occurrence in the human proteome [33] and these are the focus of the current study. While position Glu127 (PKA catalytic domain numbering used throughout the paper) of the kinase, located at the hinge that connects the two lobes, has been shown to be the source for Arg specificity at position P−3 [31], [34], [35], [36], [37], the identities and roles of kinase residues defining Arg specificities at P−2 and P−5 are more intricate. Mutational analysis [33], [38], as well as the crystal structures of several kinase-peptide complexes, confirm that different basophilic kinases use the same surface site to accommodate Arg at either the P−2 or P−5 substrate positions [39], . Positions 129, 133, 169 and a dominant acidic pair (170/230) have been implicated, but are not always sufficient for explaining the experimental P−2/P−5 Arg preferences [33], [40], [42], [43]. It appears that the P−2/P−5 Arg specificity and the interaction strength is not conferred by a readily observable sequence or structural feature, but rather by a combination of a few subtle attributes which need to be uncovered by particularly sensitive methods.

Prediction of kinase-specific phosphorylation sites is commonly based on sequence-based computational methods [44], but structure-based approaches have begun to emerge as well [45], [46], [47]. Notably, the PREDIKIN method combines structural information on specificity-determining residues with sequence information obtained from known kinase substrates [46], [47]. The currently available methods are trained on kinase and substrate sequences and rely on analogy to known complex structures. Well-suited for the detection of conserved specificity determinants between kinase subfamilies, these methods are less sensitive when specificity is dictated by kinase-unique features, and they are not aimed at supplying information on amino acid binding preferences outside the known spatial organization of the substrate/peptide complexes. Yet, such information is valuable for de novo design of protein-protein interactions inhibitors.

Computational mapping methodologies have the potential of addressing the challenge of kinase-unique binding and to specificity analyses further away from the phosphoacceptor binding region. These approaches identify the favorable binding position of a molecular probe using solely the molecular interaction field embedded in the three-dimensional structure of the protein. Consequently, a sensitive energetic description of independent functional moieties within the investigated binding environment is supplied. A variety of computational mapping methodologies have been developed, including grid based methods [48] combined with fft correlation techniques [49] and methods that employ simultaneous minimization of all probes [50]. Computational surface-mapping have been successfully used as an initial step in fragment-based drug discovery procedures [51], [52], in comparing the binding sites of different related receptors [53], and in classifying protein kinases based on their ATP-binding sites [54]. Nevertheless, since these methods are mostly designed and used for small molecules docking, they are less adequate for detecting binding positions in the context of protein-protein interactions. In the latter case, the amino acid probe is a part of a much larger molecule (protein/peptide) whose presence can modify the local dielectric environment at the probe binding site. This electrostatic shielding effect requires an appropriate treatment in order to obtain reliable scores, specific for protein-protein and protein-peptide interactions.

A specific scoring function for protein-peptide interactions is implemented in the PepSite method, which uses spatial position scoring matrices derived from a large set of protein-peptide complexes and is aimed to identify preferred amino acid binding positions on a given protein surface. By combining the predictions of single amino acid binding sites with the sequence order of the peptide, the method was shown to correctly locate the binding position of many peptides [55]. Yet, the single amino acid predictions were not tested explicitly by the authors.

Here we use ANCHORSmap [56], a recently developed computational mapping procedure specifically designed to identify binding positions of single amino acid side chains, in the context of protein-protein interactions, to study the Arg-binding preferences of representative basophilic and non-basophilic Ser/Thr kinases. ANCHORSmap consists of a specialized scoring function which was calibrated and tested for the ability to accurately position residues at protein-protein interface and to reproduce experimental ΔΔG values that were measured for alanine mutations [56].

We show that ANCHORSmap successfully discriminates between basophilic and acidophilic kinases and accurately identifies and top-ranks all P−2 and P−5 Arg-binding sites previously determined by X-ray crystallography. Furthermore, the Arg-binding positions detected for all 10 examined kinases are in line with their SCSs. A detailed examination of the Arg-binding maps produced for the different kinases, together with in-silico mutagenesis, sequence alignments and available crystal structures of kinase-peptide complexes, indicates important roles for several previously unappreciated positions and structural features in kinase catalytic domain that govern the P−2/P−5 Arg specificity.

## Results

The ANCHORSmap algorithm produces detailed binding maps of amino acid side chains on protein surfaces. The predicted binding positions (anchoring spots) are ranked by their calculated ΔG values, and adjacent anchoring spots can also be clustered into a single position to produce a sparser map of mean anchoring spots without significantly lowering the accuracy of the results [56]. In this work, the mean anchoring spots are reported unless otherwise stated, and in order to imitate a real prediction scenario, all of the calculations were performed on the unbound structures of the proteins.

Previous findings indicate that amino acids that have high propensity to form hot spots, such as Arg, Glu/Asp, Tyr, Trp and His, are also highly selective in binding to the entire protein surface [56]. As a preliminary test for the prediction sensitivity of ANCHORSmap for protein kinase surfaces, we tested the method for its ability to distinguish between basophilic and acidophilic kinases. Using both acidic (Glu) and basic (Arg) probes, the method produced a clear differential binding pattern between few representative basophilic and acidophilic kinases, indicating that it is sensitive enough for categorizing the basophilic/acidophilic nature of a given kinase without prior knowledge of its SCS (See Text S1 and Figure S1).

### The Arg-binding positions previously shown by X-ray crystallography to anchor at the −2/5 site are accurately reproduced by ANCHORSmap as top-ranking solutions

X-ray crystallography studies have shown that different kinases use the same surface site to bind Arg from either the P−2 or P−5 substrate positions. We will refer to this surface site as the −2/5 site. To the best of our knowledge, structures of kinase-peptide complexes in which an Arg residue has been shown to anchor at the −2/5 site are currently available for only four different basophilic kinases from three kinase families, defined in [26],[57]: PKA [39] and PKB [40](AGC family), PIM1 (CAMK family) and PAK4 (STE family) [58].

**Figure 1:**
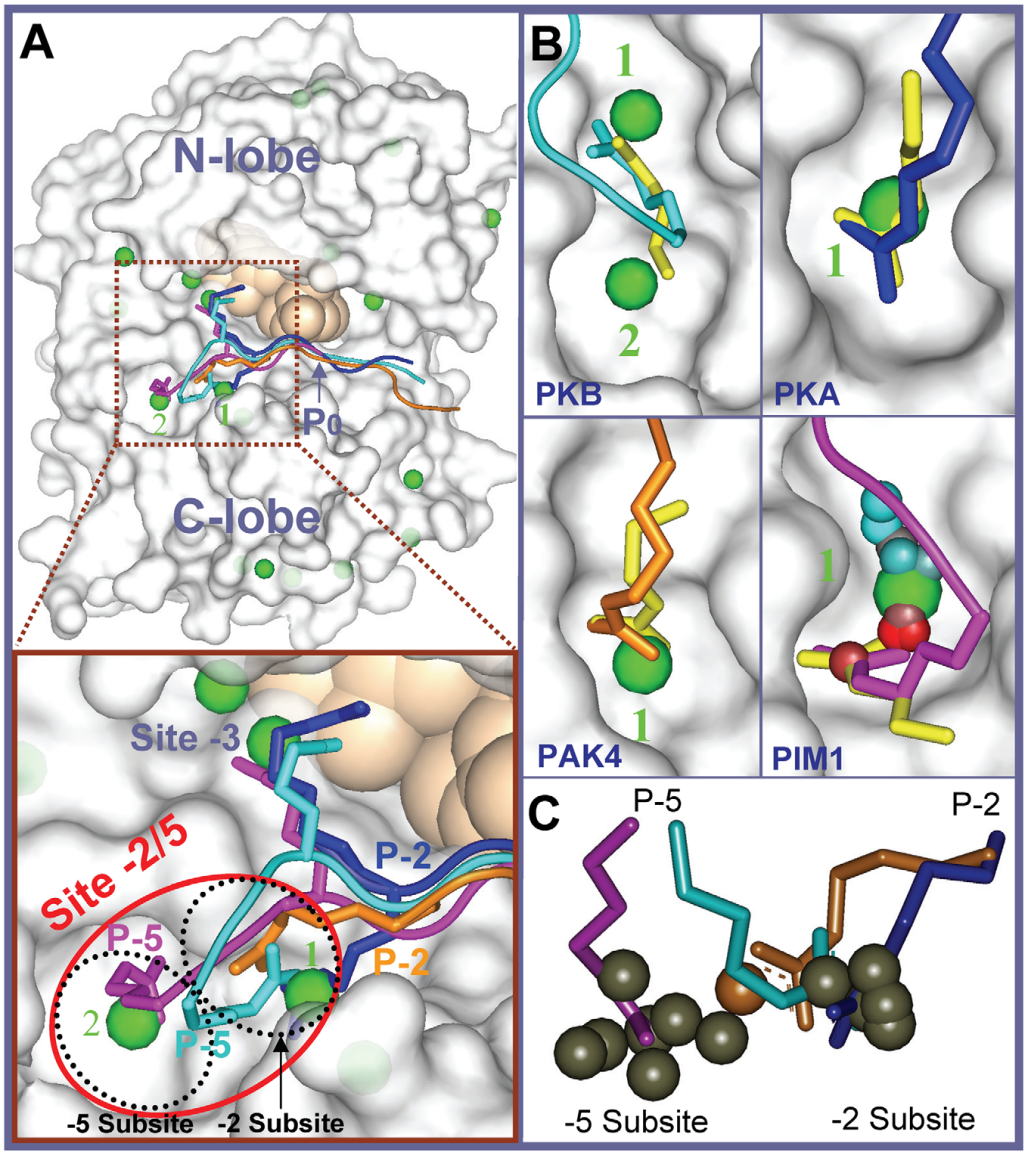
ANCHORSmap correctly identifies the Arg-binding positions at the −2/5 site that were determined crystallographically. Kinases are shown in gray surface representation, ATP is shown as light-brown spheres. Arg mean anchoring spots are shown as isolated spheres according to the position of the Arg Cζ atom and lowest RMSD positions are shown as yellow sticks. Selected rankings of mean positions are shown as green numbers. The four experimentally determined bound peptides are colored in cyan, blue, orange and magenta, for the PKB (1O6K), PKA (1JLU), PAK4 (2Q0N) and PIM1 (2BIL) kinases respectively. The peptides are presented from the most N-terminal-anchored Arg position. P−5, P−3 and P−2 Arg residues are shown as sticks. (A) An overview of the kinase surface of PKB with top 20 mean anchoring spots and a superposition of all four peptides. The brown rectangle is a magnification of the −2/5 site (red ellipsoid) region. The −2 and −5 subsites are shown as dashed black ellipsoids. (B) Experimental vs. ANCHORSmap-identified Arg-binding positions. The complexes were superimposed on the unbound kinases (gray surface). For PIM1, in addition to the mean position (green sphere), all Arg positions constituting the mean position are also shown as small spheres colored according to energetic scale from red (−14.7 Kcal/mol) to cyan (−4.2 Kcal/mol). (C) Distribution of the Arg predictions (gray spheres) between the −2 and −5 subsites. The predicted and the experimental Arg positions of PAK4 are colored orange.

Using the unbound structures of the proteins listed in Table 1, we tested the ability of ANCHORSmap to correctly reproduce their Arg-binding positions. Both detailed and mean Arg-binding maps were produced and an example from the top 20 mean Arg-binding positions detected on the entire surface of PKB can be seen in Figure 1A.

**Table 1:** Reproducing the Arg-binding positions determined by X-ray crystallography at the −2/5 site.

					Mean solution		Most accurate solution		
	Kinase family	Kinase name	Bound structure	Unbound structure	Model Rank	ΔG [Kcal/mol]	Cζ distance	Model Rank	RMSD (Å)
1	AGC	PKA	1JLU	1BKX	**1**	−11.7	1.3	2	1.6
2		PKB^a^	1O6K	3D0E	**1**(8)	−7.4(−5.1)	1.7(1.7)	9	1.3
3	CAMK	PIM1	2BIL	1XR1	**1**	−14.7	2.6	2	1.6
4	STE	PAK4	2Q0N	2CDZ	**1**	−8.3	1.6	4	1.9

The RMSD between the ANCHORSmap-identified and the experimental Arg-binding positions was calculated over all heavy atoms of the Arg residue, apart from the Cβ atom. Cζ distance - distance (Å) between the identified and the experimental Arg Cζ atoms.

a Results produced with a bound-like rotamer and unbound rotamer (in parentheses) of Glu170, see text.

Remarkably, although the search for Arg-binding positions started from thousands (∼7500) of probes initially scattered over the entire surface of each kinase, the computed positions corresponding to the experimental Arg-binding positions were ranked extremely high. For three out of four cases, the rank of the most accurate position in the detailed maps was lower than 4, and the top-ranking mean solutions coincided with the experimental Arg-binding positions in every case (Table 1). The solutions were also geometrically accurate: the average RMSD from the experimental positions was 1.6±0.3 Å, and for three out of four kinases, the top ranking mean Arg position was less than 1.7 Å from the experimental bound position (measured between the experimental and computed Arg Cζ atoms, which represent the centers of the guanidino groups). The only exception was PIM1, for which a larger distance of 2.6 Å was obtained. Examination of the entire cluster of anchoring spots that contribute to the mean position of PIM1 showed a clear tendency of the lowest-energy binding positions to accumulate in close proximity to the experimental Arg position (Table 1 and Figure 1B).

**Figure 2:**
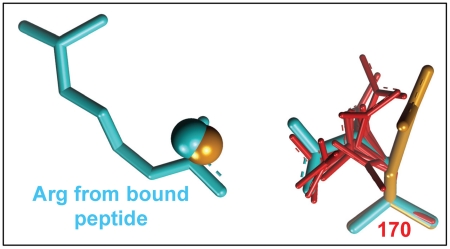
Unbound conformation of position 170 for several kinases. The structure of the PKB-peptide complex is shown in cyan stick representation. The mean anchoring spots detected for the unbound structure of PKB (3D0E) with a native (orange stick) or bound-like (cyan stick) conformation of position 170, are shown in orange and cyan CPK, respectively. The unbound conformation of position 170 for all the other kinases listed in Table 2 is colored red.

The acidic residue at position 170 of the kinases has been implicated in imposing a preference for Arg at position P−2 or P−5 of the substrate [33]. A comparison of the unbound structures of different kinases (listed in Table 2) showed that the acidic 170 residue may adopt different conformations. For the peptide-unbound PKB structure (3D0E), the conformation of Glu170 uniquely and significantly deviated from the peptide-complex structure (1O6K) and from the consensus unbound conformation observed for the other kinases (Figure 2). Thus, for PKB, both a bound-like rotamer of Glu170 (reported in Table 1) and the unbound conformation were used in the calculations. The unbound conformation of Glu170 reduced the binding affinity (by 2.3 kcal/mol) and worsened the ranking (from 1 to 8) of the correct mean solution. Nevertheless, the location of the top solution remained the same, in line with Ben-Shimon and Eisenstein's finding that mean anchoring spots are particularly useful for unbound predictions [56].

**Table 2:** Predicted Arg-binding positions in the -2/5 site correspond to SCSs.

	Kinase Family	Kinase Name	PDB code	ΔG-5 subsite	ΔG-2 subsite	Substrate Consensus Sequence (SCS)
1	AGC	**PKA**	**1BKX**	-	−11.7	R***R***XS/T [39], [85]
2		**PKB** a	**3D0E**	−6.8	−7.4	***R***XRXXS/T [86], [87]
3		**p70S6K**	**3A62**	−9.7	−4.9	***R***XRXXS/T [33], [88]
4		PDK1	3HRC	-	-	TFCGT [64]
5		PKC^b^		−6.2±0.6	−4.7±1.1	RXXS/TXRX [61], [62]
6	CAMK	**PIM1**	**1XR1**	−14.7	-	***R***XRXXS/T **,** ***R***XRHXS [70]
7		**PASK** c	**3DLS**	−8.3	−9.1	***R***XR***R***XS/T [31]
8		CAMK-II	3KK8	−4.7	-	RXXS/T [89]
9	STE	**PAK4**	**2CDZ**	-	−8.3	XXR***R***XS/T [33], [43]
10		ASK1	2CLQ	−5.7	−4.3	XX[T/Q]XT [67]

The group of -2/5 binders is emphasized in bold. Arg at positions P-2/P-5 of the SCS are indicated by bold and italics. The phosphoacceptor residues are underlined. Energies are in Kcal/mol.

a For PKB, results were produced with corrected rotation of Glu170, see text.

b Average results of four PKC isomers, alpha, betaII, iota and theta.

c Consensus sequence was determined for the yeast ortholog (Psk2).

These results are particularly striking in view of the challenging computational task. For example, we analyzed the 10 top ranking Arg positions produced by the PepSite server (http://pepsite.russelllab.org/) for each of the four kinases listed in table 1. The best result was achieved for PKB, for which PepSite solution ranked 6 was located in a distance of 4.8 Å (measured between the Arg Cζ atoms) from the experimental position. This solution corresponds to the second top ranking solution produced by ANCHORSmap for PKB, see Figure 1. For the rest of the cases tested, no reported solution was found closer than 8 Å from the experimental position.

### The Arg-anchoring spot maps correspond to Substrate Consensus Sequences (SCSs)

Six additional kinases from the AGC, CAMK and STE kinase families, for which peptide-bound structures have not been determined experimentally but unbound structures as well as experimental SCSs were available, were analyzed next: PASK, CAMK-II (CAMK family), ASK1 (STE family), p70S6K, PDK1 and PKC (AGC family). This completed the test set to 10 kinases.

Substrate-specificity studies for PKC isozymes have resulted in several, sometimes inconsistent SCS definitions [29], [38], [59], [60]. Therefore, the most frequent SCS of all PKC isozymes (**R**XX**S/T**X**R**X) [61], [62], [63] was compared to the average results obtained for the four PKC isoforms (alpha, betaII, iota and theta) for which unbound crystal structures are available.

Eight out of ten SCSs in the set contained the robust basophilic signature of Arg at P−3, but were diverse in terms of Arg-binding preferences for the −2/5 site: the set included kinases with no clear preference for Arg in either the P−2 or P−5 positions of the SCS (PKC, ASK1, CAMK-II, PDK), kinases with clear and exclusive P−2 (PKA, PAK4) or P−5 (PKB, p70S6K, PIM1) Arg preferences, and a kinase with dual P−2 and P−5 preference (PASK). Our goal was to predict the preference for Arg at the −2/5 site in general and to identify potential reasons for the preferences for −2 vs. −5 substrate positions.

#### The calculated ΔG values for Arg at the −2/5 site are in line with the existence of Arg in positions P−2 and/or P−5 of the corresponding SCS

The SCS of 6 out of 10 kinases in our set contains Arg at either the P−2 or P−5 positions (the “−2/5 binders”, highlighted in Table 2). Arg does not appear in these positions in the SCSs of the other four kinases (the “−2/5 non-binders”).

**Figure 3:**
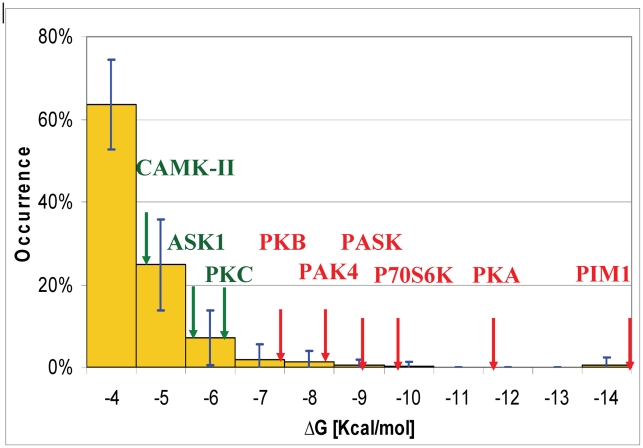
ΔG values calculated for Arg at the −2/5 site in comparison to random Arg-binding positions. The random Arg ΔG distribution is shown as orange columns and the ΔG values obtained for Arg at the −2/5 site for the 10 kinases analyzed in this study are marked with red and green arrows for the group of binder and non-binder kinases, respectively. For PDK1, no solution was detected.

Figure 3 presents the calculated ΔG values for Arg at the −2/5 site of all 10 kinases. These energies were mapped onto the average energy distribution of Arg-binding positions, as obtained from the entire surface of a random set of 20 soluble unrelated proteins (see materials and methods). The predicted ΔG values sorted in perfect agreement with the experimental data, namely, the calculated values for all of the binders were lower than the corresponding ΔGs for the non-binders. For PDK1, no Arg-binding position was detected at the −2/5 site, in line with the lack of specific preference N-terminal to the phosphoacceptor (*T*) in the SCS of PDK1 (*T*FCGT) [64].

The calculated ΔG values for the binders were found at the lowest part in the predicted energy distribution, which was occupied by only 2.5% of the cases in the random test set. This result indicated that even in terms of absolute values, the −2/5 sites of the −2/5 binders share a very unique and strong Arg-binding environment, which is distinguishable from the group of non-binders.

**Figure 4:**
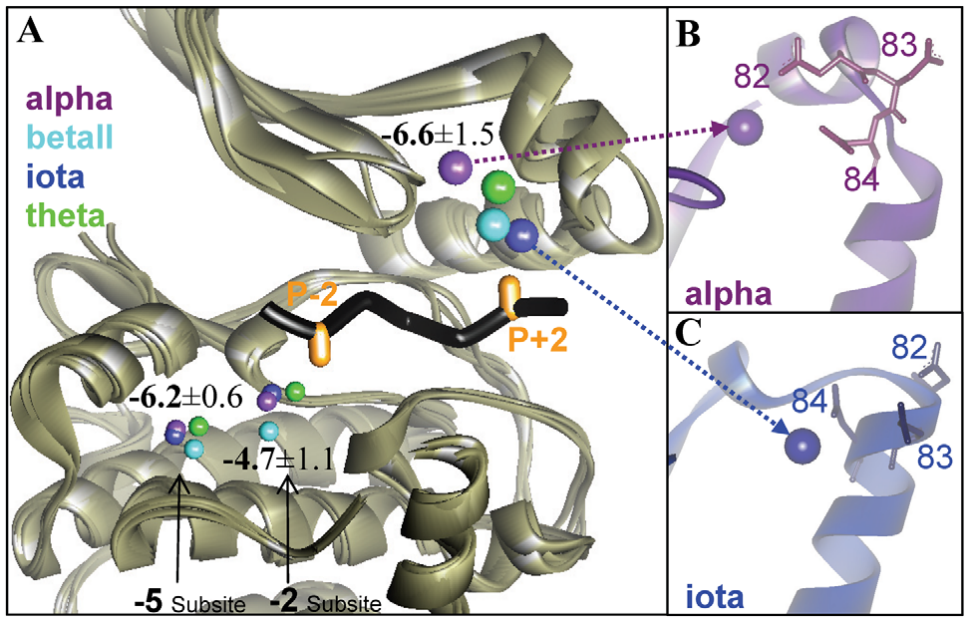
Predicted Arg-binding positions in the major binding groove of PKC isomers. The N and C lobes of each isomer (gray ribbon) were superimposed independently on the structure of the PKA-PKI complex (1ATP). (A) Viewing the predicted positions with respect to the PKI peptide. PKI (black) is presented from positions P−3 to P+3 and the P−2 and P+2 positions are emphasized in orange. The predicted Arg-binding positions (spheres that represent the Arg Cζ atom) of the four PKC isomers, alpha, betaII, iota and theta, are colored purple, cyan, blue and green, respectively. (B,C) Examples of the involvement of residues 82–84 on helix αC in Arg binding.

The strongest Arg-binding position among the −2/5 non-binder group was detected for PKC. The average calculated ΔG for the four isomers was −6.2±0.6 kcal/mol and was separated by only 1.2 kcal/mol from the weakest binding position detected for the −2/5 site binders (PKB, ΔG = −7.4 kcal/mol). This limited ΔG gap reflects some of the uncertainty regarding the ability of PKC to bind Arg at position −2 and at additional positions N-terminal to the P0 position (−4, −5, −6) [60]. Interestingly, the strongest average ΔG value (−6.6±1.5 kcal/mol) for Arg binding on the surface of PKC was detected in the region that accommodates residues C-terminal to the phosphoacceptor. This position was top-ranked for the betaII and theta isomers and ranked second for the iota isomer. For PKC alpha, the corresponding position was somewhat weaker in energy (ΔG = −5.2 kcal/mol) and it was ranked 11. Nevertheless, for all four isomers, this Arg interaction was similarly mediated by a cluster of acidic residues (positions 82–84) located on helix αC. Using statistics and an evolutionary model, Li and coworkers [42] suggested that the same surface region is adequate for accommodating the P+2 Arg that characterizes the SCS of PKC isomers (**R**XX**S/T**X**R**X) [61], [62], [63]. Indeed, the top predicted Arg-binding positions were located in very close proximity to the P+2 site (Figure 4A). Interestingly, while Li *et al.* pinpoint residues 83 and 84 as the specificity-determining residues, our results suggest that position 82 is also important for Arg binding: in PKC alpha, betaII, and theta, positions 82 and 84 dominate the Arg interactions (Figure 4B). Actually, it is only in PKC iota that a unique arrangement of the αC helix results in direct involvement of positions 83 and 84 in Arg binding (Figure 4C). Notably, the interplay between all three acidic residues is probably enabled by the flexibility of the acidic cluster region [65].

##### *Dissecting the −2/5 site into subsites that correspond to SCSs*

Is it possible to predict, based on the unbound structure of the kinase, which of the substrate positions—P−2, P−5 or both—is likely to be occupied by an Arg? Such a high-resolution prediction is first and foremost dependent on the tendency of the two substrate positions to bind at distinct subregions within the −2/5 kinase site. Superposition of the four available crystal structures containing a P−2/P−5-bound Arg at the −2/5 site showed that the Arg residues exploit two distinct surface regions within the −2/5 site (Figure 1A). However, in terms of position within the substrate sequence, the separation is less obvious. While the guanidino groups of two P−2 Arg residues (PKA, PAK4) cluster into the same subsite (referred to as −2 subsite), and the P−5 Arg of PIM1 binds in a distinct subsite within the −2/5 site (referred to as −5 subsite), in PKB, a P−5 Arg binds at the −2 subsite (Figure 1A).

For most kinases tested, ANCHORSmap detected either a single (4 kinases) or double (5 kinases) Arg-binding locations within the −2/5 site. The exception was PDK1, for which no Arg-binding position was detected (discussed earlier). Visual inspection revealed that the number of predictions is related to the shape of the −2/5 pocket. Thus, a double prediction can potentially occur in kinases with an elongated pocket, whereas a single prediction is caused by the geometrical restriction of a shorter and tighter pocket, with limited available space. For an example of predictions in elongated vs. short pockets, see PKB and PKA in Figure 1B, respectively. The Arg-binding positions detected within the −2/5 site clustered to either one of the two subsites, with seven occurrences for each subsite. A binding position was ascribed to a subsite based on the shortest distance measured between the predicted position and the subsite centers. The latter were determined by averaging the positions of the three experimentally bound Arg Cζ atoms (blue, orange and cyan) which define the −2 subsite and by the position of the single Arg Cζ atom (magenta) which defines the −5 subsite, see enlargement in Figure 1A. For PAK4, the top ranking position (perfectly compatible with the experimental Arg position observed in the PAK4-peptide complex) is shifted to be between the two subsites, yet it is closer to the −2 subsite (shown in orange in Figure 1C).

In summary, the crystal structures as well as the predicted Arg-binding positions point to the existence of two subsites within the −2/5 site that can potentially accommodate Arg from different positions of the substrate. Thus, the first essential condition for a possible discrimination between kinases with a P−2 or P−5 Arg preference is satisfied. Can such a preference be predicted?

**Figure 5:**
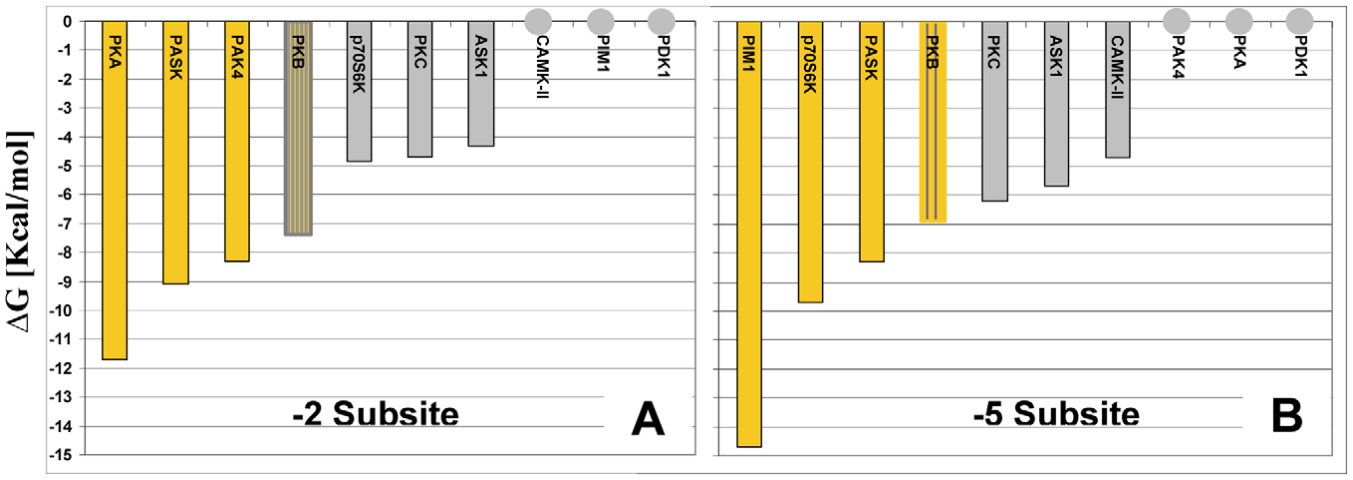
Correspondence between calculated ΔGs for Arg at the −2 and −5 subsites and their SCSs. Kinases for which the SCS contains (or does not contain) Arg at the investigated substrate positions are colored orange (gray). Mixed coloring represents the exceptional case of PKB which uses P−5 Arg to bind at the −2 subsite. (A,B) The calculated ΔG values of Arg at the −5 and −2 subsites, respectively.

The SCS of 3 out of 10 tested kinases contained Arg at position P−2. For these three kinases, the calculated ΔG values of the Arg-binding positions detected at the −2 subsite were more favorable than for the corresponding positions detected in the rest of the tested kinases (Figure 5A). Similarly, the calculated ΔG values of the positions detected at the −5 subsite were lower for 3 out of 10 kinases for which the SCS contained Arg at position P−5 (Figure 5B). A special case was PKB, for which the SCS contains Arg at P−5 but the most preferred Arg-binding position was in fact identified at the −2 subsite, with calculated ΔG of −7.4 kcal/mol. Fortunately, the crystal structures (1O6K/1O6L) of the PKB-peptide complex show that our calculations are correct, and the P−5 Arg indeed reaches the −2 subsite. Notably, the calculated ΔG at the −5 subsite is higher by only 0.6 kcal, implying that both subsites may be occupied by Arg residues.

### The predicted arg-binding positions explain both known and novel specificity-determining features at the −2/5 Site

**Figure 6:**
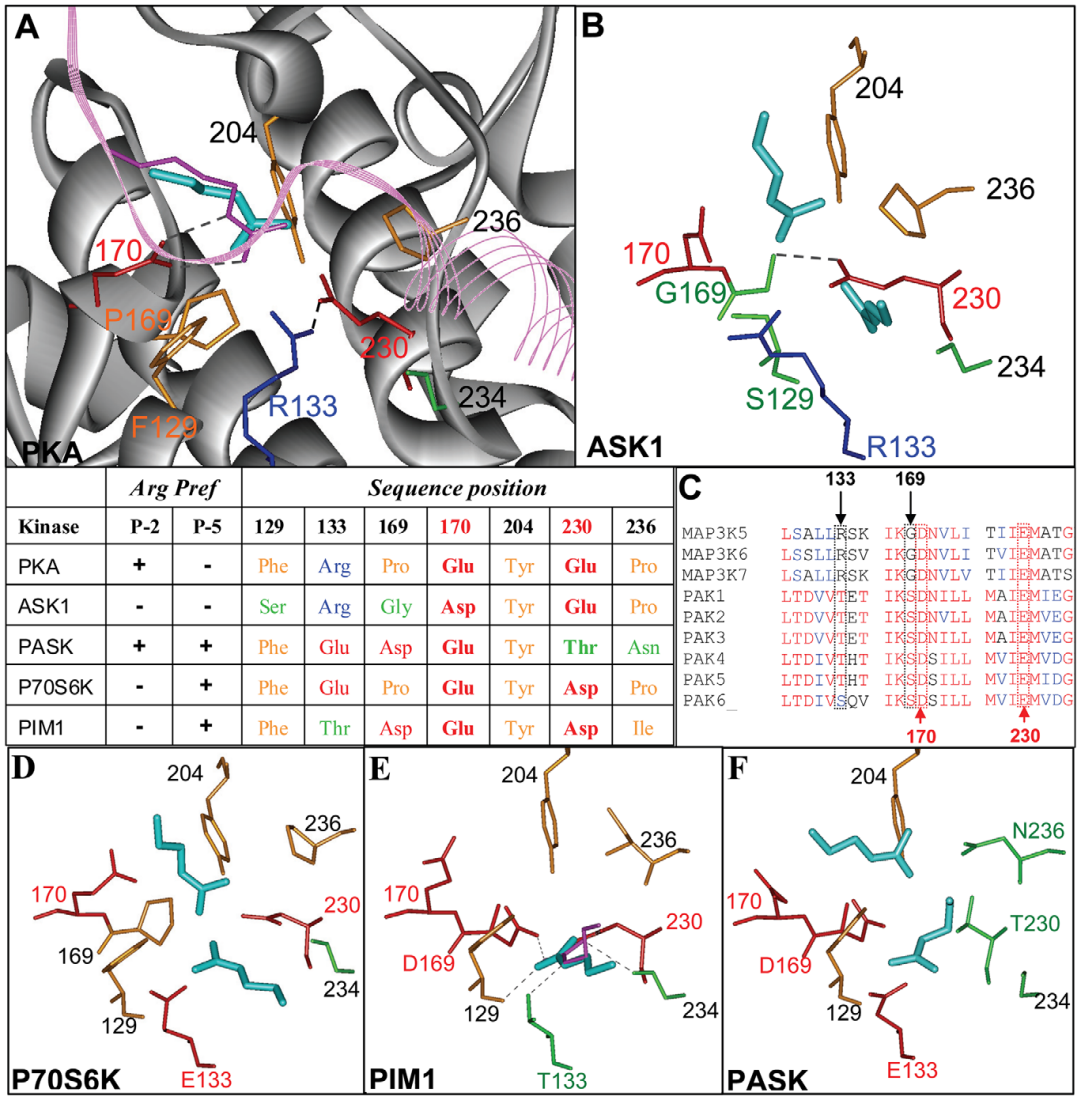
Key positions composing the −2/5 site of several kinases. Residues at positions: 129, 133, 169, 170, 204, 230, 236 and the backbone of position 234 are presented. For clarity, hydrophobic amino acids: Tyr, Trp, Ile and Pro are colored orange. Carbonyl and polar uncharged amino acids: Thr, Ser, Asn and also Gly are colored green. Negatively charged (Asp, Glu) and positively charged (Arg) amino acids are colored red and blue, respectively. Experimentally deduced H-bonds are shown as dotted gray lines. Predicted (panels A,B,D,E and F) and experimental (panels A and D) Arg-binding positions are colored in cyan and magenta respectively. For kinases for which an experimental Arg-binding position exists, the presented solution is of the lowest RMSD; otherwise, the lowest energy prediction in each subsite is presented. The table summarizes the key residue contents of all five kinases. (A) Viewing key residues at the −2/5 site of the PKA/PKI complex (1ATP). The PKI peptide is colored in cyan. (B) The predicted Arg positions and key residues at the −2/5 sites of ASK1. (C) Sequence alignment of 9 kinases from the STE family with an acidic-pair pattern. (D–F) Key residues and predicted Arg-binding positions at the −2/5 sites of P70S6K, PIM1 and PASK, respectively. For PIM1, the experimental Arg-binding position is shown in magenta.

By combining sequence alignment of key kinase residues and information extracted from SCSs, studies have suggested different residues at several sequence positions of the kinase catalytic domain (positions 129, 133, 169, 170 and 230, based on PKA numbering) as crucial for controlling the P−2/P−5 Arg specificity [31], [33], [40], [42]. The most dominant condition for attaining P−2/P−5 Arg specificity was identified by Zhu et al. [33], who showed that among the AGC, CAMK and STE kinases, P−2/P−5 Arg specificity is highly correlated with the existence of a single pair of acidic residues in positions 170 and 230. The importance of this acidic pair is illustrated in the crystal structure of the PKA/PKI complex [66], in which Arg19 (P−2) of PKI, which is anchored at the −2 subsite, is hydrogen-bonded to Glu170 and also forms a tight salt bridge with Glu230 (Figure 6A). However, it was not established whether Arg binding at the −5 subsite would also use the 170/230 acidic pair for binding. Indeed, in the crystal structure of the PIM1-pimtide complex [58], position 170 do not seems to be directly involved in the binding of the P−5 Arg. Moreover, for several kinases, the acidic pair is neither a sufficient nor an obligatory condition for attaining a strong Arg-binding preference [33], [67], and none of the currently suggested residues is able to explain the P−2/P−5 Arg preference of all kinases. We propose that this preference is most likely dictated by a delicate balance between the entire residue composition at the −2/5 site and other essential structural elements which cannot be simply detected by sequence-based methods.

To support this idea we combine the information obtained from the predicted Arg-binding positions of selected kinases analyzed in this study (PKA, ASK1, P70S6K, PASK and PIM1) with structural and sequence comparisons of key positions making up the −2/5 site (some previously suggested in the literature and others suggested here for the first time), to examine in detail the Arg-affinity-determining components at the −2/5 site. We also provide an estimation of the binding-energy contribution of selected key residues to Arg binding, by performing in-silico mutagenesis and recalculating the Arg-binding energies with ANCHORSmap. Except for one case in which Phe was replaced by Ser, the rest of the mutations were chosen such that they minimally affect the shape of the −2/5 site pocket, yet enable to determine the contribution of the residue's functional group to Arg binding. This was done by replacing charged/polar residues with hydrophobic residues of comparable size, and selecting the appropriate side-chain rotamer which would optimally mimic the native surface shape of the −2/5 site pocket. Thus Thr, Asp/Asn and Glu were replaced by Val, Leu and Met, respectively.

#### The 170/230 acidic pair is insufficient for attaining strong Arg binding at the −2/5 site of ASK1

For ASK1 (MAP3K5), the existence of the acidic pair 170/230 is insufficient for attaining a strong P−2/P−5 Arg preference [33], . A comparison between the −2/5 sites of ASK1 and PKA reveals that although the two kinases belong to different families (STE and AGC, respectively), their sites are very similar, exhibiting only two key residue differences. The first is at position 129, which is occupied by Ser in ASK1 and by Phe in PKA (Figures 6A and 6B). It was suggested [42] that Phe129 is important for PKA P−2 affinity for Arg, thus supporting the lack of Arg affinity in the case of ASK1. ANCHORSmap calculation performed on in-silico-mutated PKA showed that a Phe-to-Ser replacement at position 129 indeed reduces the predicted Arg-binding energy at the −2 subsite of PKA by 3 kcal/mol. However, the resulting ΔG value (−8.7 kcal/mol) remains very favorable and is comparable to that of other −2 subsite Arg binders (see Figure 5A).

The second difference is observed at position 169, with Pro in PKA and Gly in ASK1. Gly at position 169 frees the amide backbone to form a hydrogen bond with the essential Glu230. Consequently, the −2 subsite of ASK1 is narrower and the restricted conformation of Glu230 impairs the ability to make optimal contact (as seen for PKA) with the anchored Arg. More importantly, the constrained Glu230 cannot form a hydrogen bond with Arg133 as it does in PKA. Consequently, Arg133 of ASK1 is rotated, exposing the −5 subsite pocket, which is blocked in both the bound and unbound active structures of PKA [66], [68] (see Figure 6A). Previous ANCHORSmap calculations have shown that such a conformational change may affect the packing and dielectric environment at the −2 subsite, leading to an over 5 kcal/mol reduction in the binding affinity of Arg [56]. It has also been suggested that a non-bulky residue at position 133 dictates an Arg preference in the P−5 rather than P−2 position [40].

Sequence analysis revealed that while Pro is frequently found in position 169 of the AGC and CAMK families, at 74% and 67%, respectively, in the STE family, Pro occupies only 18% of the cases while the rest are occupied by the small residues Gly, Ala and Ser at approximately equal frequencies. Apart from ASK1, eight additional kinases in the STE family contain the 170/230 acidic-pair pattern. Six belong to the PAK1-6 group [69], and the other two are closely related to ASK1 (MAP3K6 and MAP3K7). However, only the three ASK1-related kinases contain the unique sequence combination of Gly169 and Arg133 (Figure 6C).

Since the significant structural rearrangement observed at the −2/5 site of ASK1 does not allow a reliable prediction of the effect of Pro replacement at position 169 of ASK1 by Gly-to-Pro mutation and energetic evaluation, it would be interesting to experimentally test how such a mutation affects the ability of ASK1 to prefer substrates with Arg at P−2.

The crucial effect of Arg133 and its accurate positioning in providing a suitable packing environment for Arg binding at the −2 subsite is also evident from the comparison of the −2/5 sites of PKA and P706SK, both from the AGC family. The two sites are almost identical, differing by only one Arg-to-Glu replacement at position 133. Yet, similar to ASK1, P706SK does not prefer Arg at the −2 subsite, because the positioning of Glu133, similar to Arg133 in ASK1 does not provide the required packing environment for strong Arg binding at the −2 subsite (compare Figures 6B to 6D).

Importantly, ANCHORSmap automatically captured the physicochemical differences observed at the −2 subsites of PKA, ASK1 and P70S6K as it detected a strong Arg-binding position (−11.7 kcal/mol) for PKA, as opposed to weak binding positions at the corresponding sites of ASK1 and P70S6K, with ΔG values of −4.3 and −4.9 kcal/mol, respectively.

#### Acidic pairs selected from positions 133, 169 and 230, but not from position 170, are important for Arg binding at the −5 subsite

A strong preference for Arg at P−5 is less frequently observed than at P−2 [31]. In our set, correspondence between a strong Arg-binding position at the −5 subsite and the preference for Arg at P−5 was found for three kinases, PIM1, P70S6K and PASK, with calculated ΔG values of −14.7, −9.7 and −8.3 kcal/mol, respectively.

It has been suggested that Asp at position 169 dictates Arg specificity at P−5 [31]. Such specificity characterizes the group of PIM kinases [70], which is the only group in the CAMK family (3 out of 83) that also has an acidic-pair pattern. The crystal structure of the PIM1-pimtide complex indeed shows direct involvement of Asp169 in the extensive network of five hydrogen bonds that mediate the strong peptide P−5 Arg interaction at the −5 subsite. Apart from the side chain of Asp169, the anchored Arg forms hydrogen bonds with the side chains of Thr133 and Asp230, and with the backbone carbonyl at positions 129 and 234 (Figure 6E). ANCHORSmap calculation on in-silico mutated PIM1 showed that among the three side chains, Thr133, Asp169 and Asp230, it is the latter pair of acidic residues that strongly dominates the Arg interaction, each contributing around 5 kcal/mol to the interaction, whereas Thr133 is not crucial for Arg binding (Table 3).

**Table 3:** In-silico mutagenesis highlights key positions for Arg binding.

	−2 subsite	−5 subsite
**P70S6K (WT)**	**−4.9**	**−9.7**
P70S6K (E133M)	NC	2.9
P70S6K (E170M)	NC	NC
P70S6K (D230L)	NC	2.6
**PIM1 (WT)**	**-**	**−14.7**
PIM1 (T133V)	-	NC
PIM1 (D169L)	-	5.3
PIM1 (E170M)	-	NC
PIM1 (D230L)	-	**5**
**PASK (WT)**	**−9.1**	**−8.3**
PASK (E133M)	NC	3
PASK (D169L)	4.8	1.8
PASK (E170M)	NC	NC
PASK (T230V)	NC	NC
PASK (N236L)	1.4	NC
**PKB (WT)**	**−7.4**	**−6.8**
PKB (E170M)	2	NC
PKB (E230M)	1.8	2
**PAK4 (WT)**	**−8.3**	-
PAK4 (D170L)	1.1	-
PAK4 (E230M)	3.2	-
**PKA (WT)**	**−11.7**	**-**
PKA (E170M)	3	-
PKA (E230M)	5.7	-

Energies are in kcal/mol. Calculated ΔG values of wild-type kinases are emphasized in bold and the rest of the energy values refers to the energy change associated with each mutation. Hyphen stand for cases in which no Arg binding position was detected at the investigated sub site.

NC, no significant energy change (>±1 Kcal/mol) occurred upon mutation.

P70S6K also prefers Arg at P−5. However, in contrast to PIM1, position 169 of P70S6K is occupied by Pro and not by an acidic residue. A detailed examination of the ANCHORSmap-predicted Arg-binding position detected at the −5 subsite of P70S6K revealed that the Arg interaction is mediated by the important acidic residue at position 230 (as in PIM1) combined with Glu133 (Figure 6D). Our in-silico mutagenesis analysis confirmed that these two acidic residues, Asp230 and Glu133, dominate the Arg interaction at the −5 subsite of P70S6K, with an estimated energetic contribution of −2.6 and −2.9 kcal/mol, respectively (Table 3).

Most interesting is PASK, which contains Thr instead of an acidic residue at the important position 230, but nevertheless uncharacteristically prefers Arg at both P−5 and P−2 [31]. The acidic-residue content of PASK displays a combination of PIM1 and P706SK: similar to the PIM group, it contains Asp169, and similar to P70S6K, it contains Glu133.

There is currently no complex structure of PASK with a peptide that would enable detailed analysis of the Arg interactions, but ANCHORSmap detected a strong Arg-binding position for both the −2 and −5 subsites of PASK, with calculated ΔG values of −9.1 and −8.3 kcal/mol, respectively. We could therefore analyze the interaction of the predicted Arg at the −2 subsite of PASK, as well as the estimated binding-energy contribution of each key position using in-silico mutagenesis analysis, to reveal that the lack of acidic residue at position 230 is mostly compensated for by Asp169 as well as Asn236, which are estimated to contribute −4.8 and −1.4 kcal/mol to the Arg interaction, respectively (Figure 6F and Table 3). These calculations support the novel involvement of Asp169 as well as Asn236 in the Arg preference at P−2 proposed herein.

Examination of the predicted Arg interaction at the −5 subsite of PASK showed that the lack of acidic residue at position 230 and the fact that Asp169 is already mostly involved in recognition of the P−2 Arg, are mainly compensated for by Glu133. In-silico mutagenesis analyses confirmed this observation. Together, Glu133 and Asp169 contribute approximately −5 kcal/mol to the interaction, with an estimated binding energy of −3 and −1.8 kcal/mol, respectively.

The uncommon acidic-residue content and the distinctive interaction arrangement at the −2/5 site of PASK are probably responsible for the unique dual P−2/P−5 Arg preference by this kinase.

Analysis of top-ranking Arg-anchoring spots in PIM1, P70S6K and PASK indicated that acidic residues at positions 133, 169 and 230 play a dominant role in determining Arg specificity at the −5 subsite. Nevertheless, each of the three kinases exploits a different composition of acidic pairs to attain P−5 Arg binding. PIM1, P70S6K and PASK use acidic pairs in positions 169/230, 133/230 and 133/169, respectively.

Unexpectedly, for PIM1, P70S6K and PASK, the in-silico mutagenesis analysis suggested that an acidic residue in position 170 is not essential for Arg binding at the −5 subsite. In contrast, for kinases with known Arg preference for the −2 subsite (PKB, PKA and PAK4), acidic residues in position 170, as well as in position 230, affect Arg binding at the −2 subsite, with an estimated binding-energy contribution range of 1.1–3 and 1.8–5.7 kcal/mol for positions 170 and 230, respectively (Table 3).

The observed dual involvement of both subsites in Arg binding, as well as the significant energy contribution to Arg binding measured for all kinases tested (except for PASK which contains Thr in position 230), indicate that an acidic residue at position 230 serves as a pivotal element in the recognition of Arg at the −2/5 site (Table 3). An acidic residue in position 170, on the other hand, emerges as −2 subsite-specific, and contributes less to the overall binding of Arg. These results illustrate how ANCHORSmap enables correct automated predictions of pocket preferences and of residues that are important for determining specificity, as supported by in-silico mutagenesis and repeated ANCHORSmap calculation.

## Discussion

We used ANCHORSmap, a novel computational mapping approach specifically designed for the detection of favorable binding positions of amino acid probes on the surfaces of proteins, to investigate the Arg preference determining elements in Ser/Thr protein kinase substrate-binding grooves. We focused mainly on the P−2/P−5 Arg-binding preferences that typically characterize the SCS of a particular surface region, defined by us as the −2/5 site.

Initially, we demonstrated that the ANCHORSmap method produces high-quality predictions by detecting differential binding patterns on the surfaces of representative basophilic and acidophilic kinases. This enabled successful discrimination between the two types of kinases without any prior knowledge of their SCSs. It also suggested that kinases might employ an either/or strategy in which their substrate-binding groove is optimized for binding either acids or bases, but not both. A kinome-wide analysis is needed to investigate this idea and is planned for further studies.

Importantly, using the unbound kinase structures, the method accurately reproduced and top-ranked the X-ray crystallography-determined Arg-binding positions at the −2/5 site of four different kinases; it also showed excellent correspondence between the calculated ΔG values obtained for Arg at the −2/5 site of all 10 kinases tested in this work and the preference for Arg in the experimentally determined SCSs.

In phosphorylatable peptide libraries, the SCSs of basophilic kinases emerge with a dominant signature preference for Arg at P−3 [27], [31]. However, for the group of basophilic kinases tested in this study, carrying an Arg preference at both the P−3 and P−2/P−5 positions, the Arg positions detected at the −2/5 site were almost exclusively top-ranked, pointing to the −2/5 site as the most preferred binding environment for Arg on the entire kinase surface. ANCHORSmap detected an adequate binding position for accommodation of P−3 Arg in all cases, yet both its ranking and calculated energies were significantly weaker (data not shown). However, since the kinase P−3 Arg interaction is known to involve, in some cases, the ATP molecule as well [66], [71], [72], the absence of ATP during the calculations does not allow for accurate ΔG calculation, making the comparison between the two sites difficult. It must also be taken into account that the experimentally observed dominant preference for Arg at P−3 is measured using phosphorylation activity, whereas we estimate the contribution of Arg to binding. Catalytic activity and binding affinity do not necessarily have to be correlated. A series of experiments [73] showed that the intrinsic affinities of several protein substrates to their respective kinases are weak compared to their apparent affinities measured in traditional steady-state kinetic-activity assays. Experimental studies with PKA and the protein kinase inhibitory peptide PKI support the hypothesis that the P−3 position may be important for catalysis but less important for the binding itself. It was shown that replacement of Arg19 of the inhibitory peptide (at position P−2, experimentally shown to bind at the −2 subsite (PDB code 1ATP)) by Gly reduces the inhibition by 520-fold, while similar Arg replacement at the equivalent P−3 position (Arg18) reduces the inhibition by only 90-fold [74]. Yet in substrate peptides of PKA selected for catalytic activity, Arg at position P−3 is the most dominant one [31], [33]. Moreover, the PKA-PKI complex is similarly formed with [66] (PDB code 1ATP) or without [75] (PDB code 1APM) the ATP molecule, even though in the former case, the guanidino group of the P−3 Arg is hydrogen-bonded to the ATP ribose. This indicates that the P−3 Arg is not the most crucial residue for inhibitor binding. Note that while the −2/5 site is located on the C-lobe, the P−3 Arg interacts with the N-lobe, requiring an optimal geometry of the two lobes for contact formation. Such contact may stabilize the two lobes together, enabling the accurate geometry for an appropriate catalytic activity, but might be less important for binding of an inhibitor.

The binding information obtained by ANCHORSmap is not identical to the information that can be gained from phosphorylation peptide arrays. Thus it is particularly useful in the design of kinase peptide inhibitors, as opposed to the design of optimal kinase substrates. Indeed, ANCHORSmap results were recently used to rationalize the structure-activity relations of a peptidomimetic library of novel PKB kinase inhibitors [76].

The four currently available crystal structures containing a P−2/P−5-bound Arg at the −2/5 site imply that Arg can potentially bind in two distinct subsites within the −2/5 site. However, the limited number of available complexes has made it difficult to draw a clear conclusion regarding the subsite separation inside the −2/5 site and its relation to the Arg position along the substrate sequence. The search for potential Arg-binding positions within the −2/5 site helped resolve this issue: it supplied a clear dichotomization of the predicted Arg positions between two separate subsites, −2 and −5, each with a different set of predicted Arg positions that usually corresponded to the location of Arg in the P−2 and P−5 positions of the SCS, respectively.

Finally, we used the predicted Arg-binding positions together with structural, sequence and *in-silico* mutagenesis analysis of key residues, to explain known and novel structural and sequence specificity-determining features that govern the Arg interaction at the −2/5 site. The analysis showed that in many cases, the interaction strength is underlined by a delicate balance between several attributes of the binding site, architectural and chemical, which cannot be simply obtained from sequence alignment comparisons, but emerge automatically from the ANCHORSmap-predicted positions and accompanying ΔG values. This is due to the fact that ANCHORSmap utilizes the information embedded in the structure of the protein, capturing subtle changes in the physicochemical properties of the binding site. The predicted positions were then used to trace back the role of individual key residues and structural features that determine preference toward Arg in particular substrate positions. This methodology provided several new hypotheses regarding Arg specificity-determining elements at the −2/5 site, which can be further tested experimentally. We suggest that the inability of ASK1 to strongly bind Arg, in spite of the existence of the 170/230 acidic pair, can be explained by a unique sequence composition that affects the architectural arrangement at the −2/5 site of ASK1. We could therefore hypothesize that the preference for a P−5 Arg is not governed by the 170/230 acidic pair, as it is for P−2 Arg and as was previously assumed, but can be governed by several different pairs of acidic residues, selected from positions 133, 169 and 230, whereas position 170 affects only the binding of Arg at the −2 subsite. Acidic residue at position 230 on the other hand, serves as a pivot element in the recognition of Arg at both subsites.

Computational mapping of amino acid-anchoring spots on kinase surfaces can provide testable hypotheses regarding kinase specificity and peptidomimetics affinity [77] and may be used as input for anchor-driven peptide-docking [14] to predict 3D structures of kinase-peptide complexes. It is therefore expected to promote our understanding of kinase regulation and expand the possibilities for the design of kinase-specific signaling modulators.

A compendium of peptide-anchoring sites obtained in this work is available upon request, providing a basis for the development of novel kinase modulators for biochemical research.

## Materials and Methods

### Producing binding maps with ANCHORSmap

#### The ANCHORSmap method

Briefly, the ANCHORSmap algorithm [56] consists of two stages: (i) a geometry-based step, in which subpockets that can accommodate single amino acid side chains are detected on the surface of the protein and amino acid probes are scattered near them. This produces a non-random yet exhaustive distribution of thousands of probes over the entire protein surface; (ii) an energy-based step in which the positions of probes, initially scattered, are optimized by several cycles of energy minimization and clustering. The minimizations are performed with the Gromacs software, version 3.3.3 [78], employing the united atoms gromos96 43a1 force field [79]. The binding energies of the probes (ΔG_p_) are estimated by an empirical scoring function adjusted for the context of protein-protein interactions: The scoring function free parameters were optimized and statistically validated using calculated versus experimental ΔΔG values of 57 alanine mutations (ΔΔG = ΔG_Ala_−ΔG_p_) of interface residues.

The scoring function consists of a corrected van der Waals (vdW) energy term (V′), a corrected and weighted (λ_e_) electrostatic term (E′) and a weighted (λ_s_) desolvation energy term, of the probe (−S_p_) and of the anchoring cavity (−S_cav_) (eq. 1).
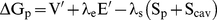
(1)
*The Corrected vdW* (V′) is normalized as suggested by Pan *et al* [80]. The Gromacs vdW energy (V) is divided by *N* ^α^, where *N* is the number of non-hydrogen atoms of the probe and α is a free parameter (eq. 2).
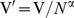
(2)
*The electrostatic energy* computed with Gromacs (E) is corrected (E′) to include the possible changes in the local dielectric environment induced by a hypothetical protein bound to the probe (eq. 3).
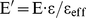
(3)Thus, an effective dielectric coefficient, ε_eff_, is calculated which takes values between ε (1.5 as in the Gromacs minimizations) and ε_max_ (free parameter) and represents the local dielectric binding environment (eq. 4).
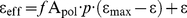
(4)The effective dielectric coefficient is estimated as the fraction of polar surface area that remains accessible in the bound probe (*f*A_pol_) multiplied by the probability that this polar area is buried in a hypothetical protein-protein interface (*p*). The probability *p* rises exponentially as the exposed surface area of the bound probe (A) grows, but it is limited to *p*≤1 (eq. 5).
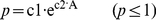
(5)The fitted values of the C1 and C2 constants are 0.0024 and 0.167, respectively. Hence, ε_eff_ for a probe that is deeply buried in a surface pocket (low *p*) is close to ε, whereas a probe that is only partially buried feels higher dielectric shielding.

*The solvation energy* (S_p_ or S_cav_) is estimated as the conventional sum over the product of atomic solvation parameter and solvent exposed area.

The above procedure produces a very detailed map of anchoring spots which is usually unnecessary when searching the entire surface of a protein. Therefore the algorithm enables averaging a cluster of adjacent anchoring spots into a single mean position, to produce a sparser map of mean anchoring spots. The averaging is weighted by the ΔG of each probe in the cluster, giving more weight to predictions with lower ΔG, and the associated energy of the mean position is set to the lowest ΔG in the cluster.

In this work, we used the default parameters of ANCHORSmap as previously described by [56] using a RMSmin of 3 Å to produce the map of mean anchoring spots.

#### Kinase structure preparation

Only kinase structures for which all the residues surrounding the −2/5 site region are resolved were used for the analysis. Kinase coordinates were obtained from the Protein Data Bank (PDB) [81]. Hetero atoms were excluded before the ANCHORSmap calculations.

### Superimposing the −2/5 Sites of different kinases

Superposition of the −2/5 site was used for probe RMSD calculations and for the comparison between the −2/5 sites of different kinases. Superposition was performed over the Cα atoms of the following list of residues surrounding the −2/5 site: 128–136, 168–170, 201–204, 230, 234–236 (PKA numbering).

### The random set of proteins

The random set of proteins consisted of 20 soluble medium-sized (255–280 amino acids), structurally and functionally unrelated structures, sharing sequence identity of less than 25%, which were taken from the PDB-REPRDB database [82]: http://mbs.cbrc.jp/pdbreprdb-cgi/reprdb_menu.pl. The list of structures are: 1BKC, 1G6H, 7YAS, 1QGI, 1P1X, 1MOO, 1QH5, 1UWC, 1JFR, 2A14, 1MML, 1ARB, 1J1M, 1AKO, 1VIN, 2A3U, 1JOV, 1NPY, 2HVM, 1IC6.

### Sequence analysis

Kinase sequences were retrieved from KinBase (http://kinase.com). Only sequences of the catalytic domain of human kinases were included in the analysis. Sequence alignment was performed using the T-coffee server [83], [84].

### *In-silico* mutagenesis

Apart from the Phe129Ser replacement in PKA, in which the rotamer of the corresponding Ser in ASK1 was used, for the rest of the mutations, the rotamer which optimally mimicked the native surface shape of the −2/5 site pocket was selected by visual inspection. The selected rotamers were then subjected to a short (20-step) steepest descent energy minimization to remove steric clashes with the protein. Side-chain replacements, rotamer selections and energy minimizations were performed with built-in tools of the Discovery Studio package V2.5.

## Supporting Information

Figure S1 **Differential anchoring spot mapping for basophilic and acidophilic kinases.** (A) Top-ranking binding positions detected for Glu (red) or Arg (blue) probes at a distance shorter than 10 Å from the substrate-binding region of the acidophilic kinases GSK3 (1O9U) and CK2 (3H30), and the basophilic kinases PKA (1BKX) and PAK1 (3FY0). Each column represents a single binding position. For distance measurement, see text. (B) Viewing the distribution of the 10 top-ranking Glu and Arg predictions on the entire surfaces of CK2 and PAK1. The position of the PKI peptide (green line), presented here with only three amino acids on each side of the P0 position (green sphere) and the position of the ATP molecule (brown spheres) were determined by superposing the structure of the PKA-PKI complex (1ATP) on each kinase. For each kinase, the top 10 mean anchoring spots detected for the Arg (represented by the Arg Cζ atom) and Glu (represented by the Glu Cδ atom) probes are shown as blue and red spheres, respectively. Note that some of the probes are invisible as they are located on the back side of the protein. Black arrows mark the corresponding binding positions appearing in panel A.
(TIF)

Text S1 **Determining whether a kinase is basophilic or acidophilic.**
(DOC)
