# Supplementary figures and images for: Deciphering the Arginine-Binding Preferences at the Substrate-Binding Groove of Ser/Thr Kinases by Computational Surface Mapping
Source: PLoS Comput Biol. 2011 Nov 17;7(11):e1002288. doi: 10.1371/journal.pcbi.1002288 (PMC3219626; doi:10.1371/journal.pcbi.1002288)

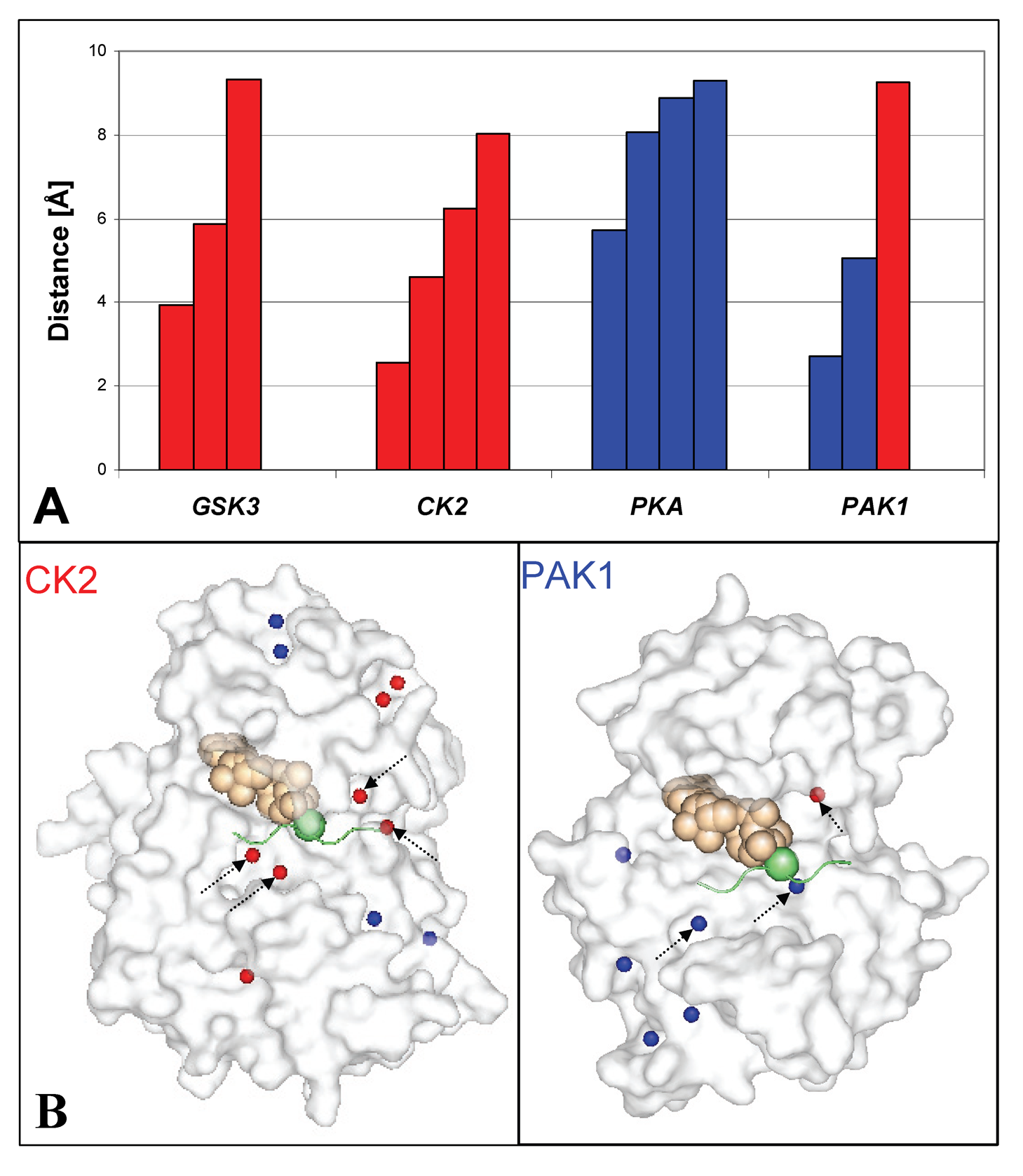

Supplement: Figure S1 — Differential anchoring spot mapping for basophilic and acidophilic kinases. (A) Top-ranking binding positions detected for Glu (red) or Arg (blue) probes at a distance shorter than 10 Å from the substrate-binding region of the acidophilic kinases GSK3 (1O9U) and CK2 (3H30), and the basophilic kinases PKA (1BKX) and PAK1 (3FY0). Each column represents a single binding position. For distance measurement, see text. (B) Viewing the distribution of the 10 top-ranking Glu and Arg predictions on the entire surfaces of CK2 and PAK1. The position of the PKI peptide (green line), presented here with only three amino acids on each side of the P0 position (green sphere) and the position of the ATP molecule (brown spheres) were determined by superposing the structure of the PKA-PKI complex (1ATP) on each kinase. For each kinase, the top 10 mean anchoring spots detected for the Arg (represented by the Arg Cζ atom) and Glu (represented by the Glu Cδ atom) probes are shown as blue and red spheres, respectively. Note that some of the probes are invisible as they are located on the back side of the protein. Black arrows mark the corresponding binding positions appearing in panel A. (TIF) [file pcbi.1002288.s001.tif]
