# Supplementary material for: Deciphering the Arginine-Binding Preferences at the Substrate-Binding Groove of Ser/Thr Kinases by Computational Surface Mapping
Source: PLoS Comput Biol. 2011 Nov 17;7(11):e1002288. doi: 10.1371/journal.pcbi.1002288 (PMC3219626; doi:10.1371/journal.pcbi.1002288)
Supplement: Text S1 — Determining whether a kinase is basophilic or acidophilic. (DOC) [file pcbi.1002288.s002.doc]

**Determining Whether a Kinase Is Basophilic or Acidophilic**

Two basophilic kinases (PKA and PAK1) and two acidophilic kinases (GSK3 and CK2) were selected, and for each (unbound) structure, an anchoring map was produced using representative basic (Arg) and acidic (Glutamic acid, Glu) probes. We expected to identify favorable Arg- and Glu-binding sites near the substrate-binding regions of the basophilic and acidophilic kinases, respectively. The proximity of the predicted probes to the substrate-binding region was determined by superimposing the structure of the PKA-PKI (a protein kinase inhibitory peptide) complex (1ATP) on each tested kinase, and measuring the shortest distance available between the predicted probe and any backbone atom of the PKI peptide (positions P-3 to P+3). Binding positions with a measured distance smaller than 10 Å were considered to be near the substrate-binding region.

Figure S1A presents Glu- and Arg-binding positions detected around the substrate-binding region of each kinase from the 10 top-ranking predictions detected on the entire surface of each kinase catalytic domain and for each probe type. For viewing examples of the 10 top-ranking predictions detected on the entire surfaces of CK2 and PAK1, see Figure S1B

A clear differential binding pattern was observed between basophilic and acidophilic kinases. For the acidophilic kinases GSK3 and CK2, no top-ranked Arg-binding positions were detected around the substrate-binding region, whereas three and four high-ranking positions were observed for Glu, respectively. For the basophilic kinases, an opposite binding pattern was observed, with four and two high-ranking Arg-binding positions detected around the substrate-binding region of PKA and PAK1, respectively. Similar results were obtained using the Arg probe compared to an Asp probe (data not shown). For PAK1, a single acidic binding position was detected at a distance of 9.3 Å (Figure S1A&B).

This result indicated that anchoring maps produced for unbound structures are sensitive enough to categorize the basophilic/acidophilic nature of a given kinase without prior knowledge of the consensus sequence of the substrate.
